# Humoral cross-reactivity between Zika and dengue viruses: implications for protection and pathology

**DOI:** 10.1038/emi.2017.42

**Published:** 2017-05-10

**Authors:** Lalita Priyamvada, William Hudson, Rafi Ahmed, Jens Wrammert

**Affiliations:** 1 Department of Pediatrics, Division of Infectious Disease, Emory University School of Medicine, Atlanta, GA 30322, USA; 2 Emory Vaccine Center, Emory University School of Medicine, Atlanta, GA 30322, USA; 3 Department of Microbiology and Immunology, Emory University School of Medicine, Atlanta, GA 30322, USA

**Keywords:** antibody-dependent enhancement, cross-protection, cross-reactive antibodies, DENV, ZIKV

## Abstract

Zika virus (ZIKV) is a re-emerging mosquito-borne flavivirus that has recently caused extensive outbreaks in Central and South America and the Caribbean. Given its association with Guillain–Barré syndrome in adults and neurological and ocular malformities in neonates, ZIKV has become a pathogen of significant public health concern worldwide. ZIKV shares a considerable degree of genetic identity and structural homology with other flaviviruses, including dengue virus (DENV). In particular, the surface glycoprotein envelope (E), which is involved in viral fusion and entry and is therefore a chief target for neutralizing antibody responses, contains regions that are highly conserved between the two viruses. This results in immunological cross-reactivity, which in the context of prior DENV exposure, may have significant implications for the generation of immune responses to ZIKV and affect disease outcomes. Here we address the issue of humoral cross-reactivity between DENV and ZIKV, reviewing the evidence for and discussing the potential impact of this cross-recognition on the functional quality of antibody responses against ZIKV. These considerations are both timely and relevant to future vaccine design efforts, in view of the existing overlap in the distribution of ZIKV and DENV and the likely spread of ZIKV to additional DENV-naive and experienced populations.

## ZIKA VIRUS EMERGENCE AND CHANGING CLINICAL FEATURES

Zika virus (ZIKV) is a mosquito-borne flavivirus that was first discovered in 1947 in the Zika forest in Uganda.^1^ The virus was isolated from a febrile rhesus macaque through a yellow fever surveillance network in the area. A year later, ZIKV was also isolated from *Aedes* mosquitoes from the same forest, pointing to a potential sylvatic transmission cycle involving non-human primates and mosquitoes.^1, 2^ The first confirmed human ZIKV case was a laboratory-acquired infection reported in Uganda in 1964^3^ following which sporadic cases of natural human infection were identified in Nigeria^4, 5^ and Indonesia.^6^ However, serological data suggests a wider geographical distribution, as seroprevalence for ZIKV antibodies has been documented in several additional countries spanning South and Southeast Asia and Africa,^7, 8^ as well as in Uganda as early as 1952.^2^

The first significant human outbreak of ZIKV outside these areas occurred on the Yap Island of Micronesia in 2007, which was marked by 49 confirmed infections, 131 probable or suspected cases and an estimated ZIKV infection rate of 73%.^9, 10^ Thereafter in 2013, the virus caused a larger epidemic in French Polynesia, with estimates ranging between 8500 and 19 000 suspected infections.^11, 12^ Until 2013, symptomatic ZIKV infections were primarily associated with mild illness involving fever, rash, myalgia, arthralgia and conjunctivitis.^7, 9^ However, during the French Polynesia outbreak, many ZIKV patients presented with severe clinical manifestations including Guillain–Barré syndrome, which required hospitalization and medical interventions.^13, 14^

In 2015, ZIKV was discovered to have spread to Brazil,^15, 16, 17^ which initiated the largest ZIKV epidemic known to date. Since its emergence in Brazil, cases of autochthonous ZIKV transmission have been reported in nearly 50 additional countries and territories in the western hemisphere,^18^ including the United States.^19^ In addition, ZIKV infections in the Brazilian outbreak have been linked to complications in pregnancy, and severe ocular and neurological deformities in neonates born to ZIKV-infected mothers including microcephaly.^20, 21, 22^ Besides the striking increase in the incidence of microcephaly reported concurrently with the ZIKV outbreak,^23^ the presence of ZIKV in brain tissues of aborted microcephalic fetuses,^24, 25^ as well as in the amniotic fluid of pregnant mothers of microcephalic fetuses^26^ demonstrate a causal relationship between ZIKV infection and this devastating developmental defect.^27^

Accordingly, ZIKV has now emerged as one of the most critical arboviruses and is a significant public health concern worldwide. Given the overlapping presence of DENV in a majority of ZIKV epidemic regions,^18, 28, 29^ there is a pressing need to better understand the extent and characteristics of DENV–ZIKV immunological cross-reactivity. Further, the potential impact of this cross-reactivity on the protective efficacy of ZIKV-induced antibody responses warrants careful investigation.

## GENETIC AND IMMUNOLOGICAL RELATEDNESS BETWEEN ZIKV AND DENV

### Structural similarities and sequence conservation

**Figure 1:**
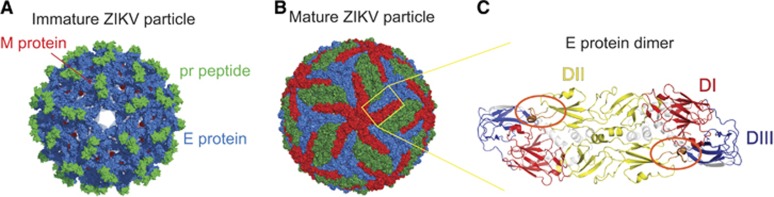
The ZIKV particle and E protein dimer. Cryo-EM surface structures of (**A**) immature (PDB 5U4W)^31^ and (**B**) mature (PDB 5IRE)^30^ ZIKV. The E protein dimer is highlighted in a yellow box. (**C**) The ZIKV E protein dimer colored by its domain, EDI: red, EDII: yellow and EDIII: blue.^30^ The fusion loop is circled in orange. All structural figures in (**A–C**) were created using PyMol (Schrödinger LLC).

**Figure 2:**
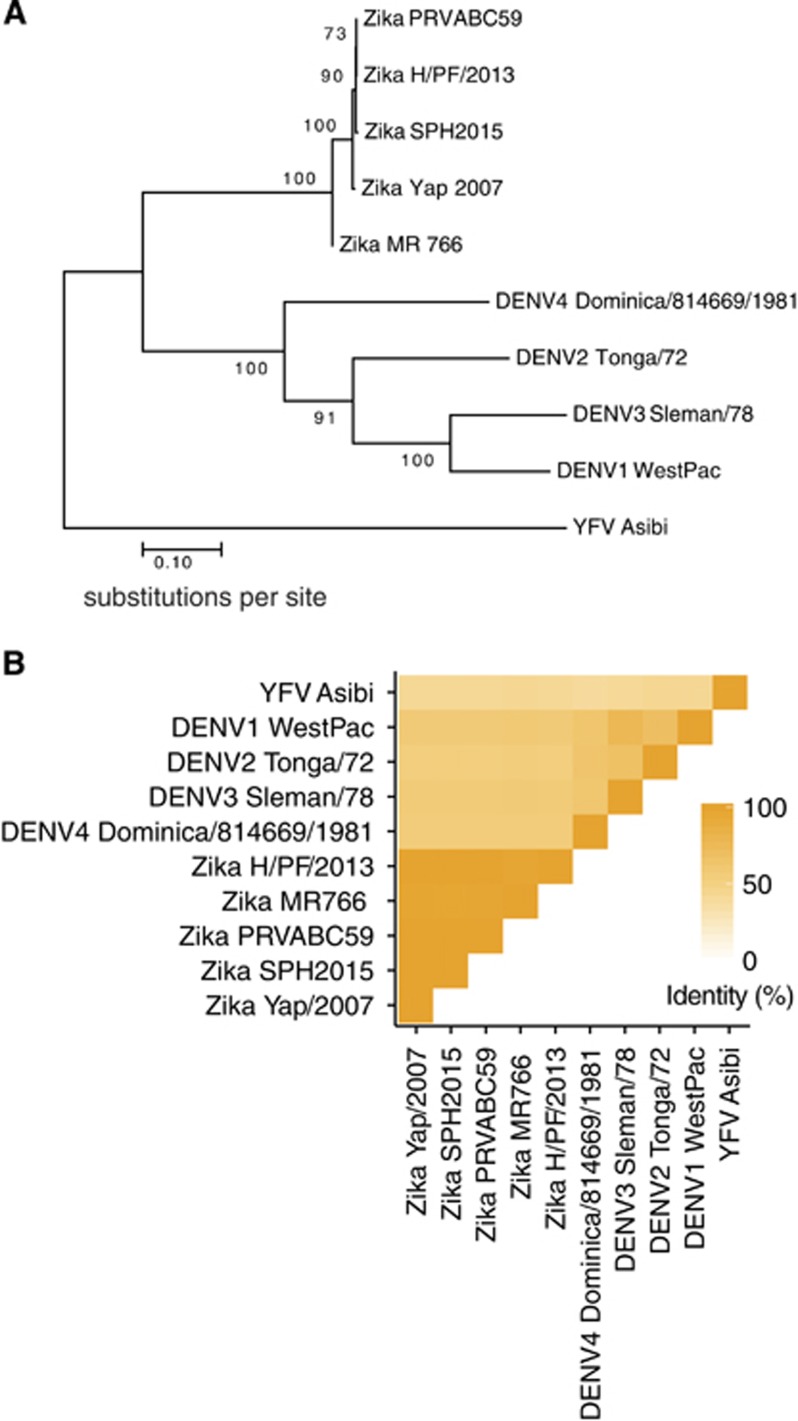
ZIKV and DENV E proteins share considerable sequence identity. (**A**) Phylogenetic tree, showing relatedness based on E protein sequence, created using MEGA7.^67^ The evolutionary history between the viruses was inferred by using the maximum likelihood method based on the JTT matrix-based model.^68^ The percentage of trees in which the associated viral sequences clustered together is shown next to the branches. Branches are drawn to scale, with lengths measured in the number of substitutions per site. (**B**) Heat map showing E protein sequence identity, generated with ggplot2 in R.^69^ Sequences were aligned in Geneious version 6.1.^70^ For (**A**) and (**B**), the ZIKV strains analyzed and their GenBank accession numbers are: PRVABC59: KU501215, MR766: AY632535, H/PF/2013: KJ776791, Yap/2007: EU545988 and SPH2015: KU321639. The DENV strains are DENV1 WestPac: U88535, DENV2 Tonga/72: AY744147.1, DENV3 Sleman/78: AY648961.1 and DENV4 Dominica/814669/1981: AF326573.1. In addition, the YFV strain Asibi: KF769016.1 was also included as an outgroup in the sequence analyses above.

ZIKV is a member of the *Flaviviridae* virus family. Its positive sense, single-stranded RNA genome is contained within a nucleocapsid core that is surrounded by an outer envelope made up of two structural proteins: envelope (E) and pre-membrane (prM). The cryo-EM structure of ZIKV reveals that the virus has a nearly identical organizational structure to DENV, including the characteristic herringbone arrangement of E protein head-to-tail homodimers on the virus surface^30, 31^ (Figures 1A and 1B). In addition to structural similarities between the viral particles, the main targets for antibody responses in dengue infections, namely E, prM and the non-structural protein NS1, share substantial amino-acid sequence identity between ZIKV and DENV.^30, 32, 33, 34^ The considerable structural and genetic relatedness between ZIKV and DENV (Figure 2) has been hypothesized to cause immunological cross-reactivity between these closely related viruses, which may make diagnosing patients challenging as well as potentially impact protective/pathologic immune responses to these infections.

The E protein is involved in receptor binding, fusion and viral entry, and is a major target for neutralizing antibody responses in flaviviral infections. The crystal structure of ZIKV E shows that like other flaviviral E proteins, it contains three E protein domains (EDs): a central β-barrel-shaped domain I (EDI), a finger-like domain II (EDII) and a C-terminal immunoglobulin-like domain III (EDIII). The viral fusion peptide is located at the tip of EDII, and lies shielded by EDI and EDIII from the other monomeric subunit within the E protein dimer^35^ (Figure 1C). In the context of DENV infection, EDIII was shown to be an important target for type-specific potently neutralizing antibodies in mice.^36, 37^ However, serum depletion experiments reveal that EDIII-specific antibodies make up a small proportion of human DENV-immune serum.^38, 39^ In contrast, the fusion loop region, containing the fusion loop epitope (FLE), appears to be more immunodominant and is a target for less potent but highly cross-reactive antibodies.^40^ Another target of significant interest is termed the envelope dimer epitope (EDE).^40^ EDE antibodies, directed toward epitopes that span across the E dimer interface, have been shown to be conformationally sensitive and broadly neutralizing. These dimer-dependent epitopes are part of a novel and growing class of complex, quaternary epitopes that are only present in the intact virion and not the monomeric form of E.^40, 41, 42, 43, 44^ Recently, mAbs generated from memory B cells (MBCs) of ZIKV-infected patients were shown to target virus-specific EDIII as well as the highly conserved fusion loop, among other sites.^33, 45, 46^ In addition, EDE-specific mAbs derived from DENV-infected patient MBCs potently cross-neutralized ZIKV.^47, 48^ These findings demonstrate the relevance of FLE, EDE and EDIII epitopes in ZIKV antibody responses. However, additional studies are required to determine the contribution of such antibodies to the overall protective capacity of ZIKV-induced humoral immunity.

### Evidence of immunological cross-reactivity

During the Yap State outbreak, suspected ZIKV cases were tested for serum binding and neutralizing (PRNT_90_) titers to ZIKV and other flaviviruses including DENV.^9, 10^ Most patients tested were categorized as flavivirus pre-immune due to the presence of cross-reactive IgG in their acute-phase (<10 days after symptom onset) sera. A majority of these presumed secondary flavivirus cases showed measurable DENV PRNT_90_ serum titers.^10^ Although ZIKV was the only detectable circulating virus during the outbreak (as stated in Lanciotti *et al*.,^10^), DENV infections had been previously reported on the island.^49^ Similarly, during the French Polynesia outbreak, cross-reactive serum titers against DENV were observed in ZIKV-probable cases.^14^ This may have been in part due to the co-circulation of DENV1 and DENV3 in French Polynesia at the time of its ZIKV outbreak.^12^ In addition, the country has experienced several dengue epidemics,^50, 51, 52^ and moreover, serological surveys in 2011–2013 indicated that nearly 80% of the adult population was DENV seropositive.^53^ Consequently, given the possibility of prior/concomitant exposure to DENV, the presence of cross-reactive antibody titers in patients from the Oceania outbreaks was not entirely unexpected.

More recently, several studies have addressed the issue of DENV–ZIKV immunological cross-reactivity by testing sera from ZIKV-infected individuals against DENV, or dengue sera against ZIKV. In one such study, sera from both DENV-naive and DENV pre-immune ZIKV patients strongly bound to ZIKV as well as DENV, with cross-reactive antibodies targeting both E and NS1 proteins.^33^ The four patient samples in this study were from primary ZIKV cases, where infection occurred during travel to ZIKV-afflicted areas. Similarly, studies examining secondary dengue sera from endemic regions have shown cross-reactivity to ZIKV, in both binding and neutralization of the virus.^32, 47, 48^

The studies described above illustrate ample cross-reactivity between ZIKV sera and DENV and vice versa. However, such analyses of polyclonal sera alone may not reveal the origin of cross-reactive antibody responses, or the relative proportion of type-specific versus cross-reactive antibodies. Especially in flavivirus-experienced populations, the possibility of multiple independent pools of antibodies contributing to the apparent serum cross-reactivity, rather than one common pool that recognizes both DENV and ZIKV also cannot be easily ruled out. This issue has been addressed by functional studies of mAbs generated from dengue patient plasmablasts. Our group and others have demonstrated that mAbs generated from *in vivo* activated, single-cell-sorted plasmablasts isolated during DENV infection can bind and neutralize ZIKV.^32, 48^ As the source of the mAbs analyzed was plasmablasts specifically activated in response to DENV infection, these studies conclusively show that dengue-induced antibodies can cross-react to a heterologous virus, ZIKV. Additionally, mAb panels generated from MBCs of DENV-naive primary ZIKV patients have also been tested against DENV antigens to demonstrate ZIKV–DENV dual-reactivity at the single-cell level.^33^

## IMPACT OF CROSS-REACTIVE HUMORAL IMMUNITY

### Key targets for cross-reactive human antibody responses

The surface glycoproteins E and prM, and the non-structural protein NS1 have been identified as the main antigenic targets for human B-cell responses in DENV infections.^54, 55, 56, 57, 58^ Whereas studies examining convalescent patients have shown the abundance of prM and NS1-specific MBCs,^54, 56, 57, 58^ focused analyses of dengue plasmablast responses demonstrate that acute-phase antibodies are largely directed to the E protein.^40, 59, 60^ Dengue-induced B-cell responses are dominated by antibodies that are cross-reactive to multiple serotypes, with a minor proportion exhibiting serotype-specific activity.^54, 56, 57, 58, 60^ Recently, panels of mAbs from ZIKV-infected patients have been characterized to study the functional properties of ZIKV antibodies.^33^ Antibodies generated from the MBCs of primary ZIKV patients included both ZIKV-specific as well as DENV cross-reactive mAbs. The subset of NS1 mAbs was largely ZIKV-specific despite the high cross-reactivity displayed by sera from the same donors. In addition, mAbs that bound EDIII, or whole virus but not recombinant E protein, were highly ZIKV-specific and potently neutralizing *in vitro*. In contrast, mAbs that were presumably EDI/EDII-specific, evident by their lack of binding to EDIII but recognition of the complete E protein, displayed cross-reactive binding to DENV but poorly neutralized ZIKV.^33^

Dengue patient plasmablast and MBC-derived mAbs have also been tested for cross-reactive binding and neutralization to ZIKV.^32, 33, 47, 48, 61^ Although binding to the virus was more broadly reported, the potent cross-neutralization of ZIKV by dengue-induced mAbs appears to be a relatively restricted phenotype.^32, 48, 61^ Antibodies directed to the highly conserved and immunodominant FLE poorly neutralized ZIKV *in vitro*.^61^ In contrast, the dimer-dependent EDE-specific mAbs were found to neutralize ZIKV potently.^47, 48, 61^ The recognition and potent neutralization of ZIKV by EDE antibodies suggests that quaternary and other complex epitopes may be important antibody targets in the ZIKV immune response. Mapping the epitopes of additional ZIKV-neutralizing mAbs may reveal novel antigenic sites critical for protection and also inform future vaccine development efforts.

### Antibody-dependent enhancement

Alongside their protective potential, antibody responses in dengue and other flaviviral infections have also been implicated in exacerbating disease. Studies have shown that infecting Fcγ-receptor (FcγR)—expressing cells in the presence of antibodies from flavivirus-immune donors—can significantly increase the rate of infection.^62^ This phenomenon, termed antibody-dependent enhancement (ADE), is said to occur when cross-reactive antibodies present at sub-neutralizing concentrations facilitate the uptake of virions by permissive cells, thereby enhancing infection. Rather than inhibiting viral infection, the immune complexes formed between such antibodies and viral particles attach to cells and are internalized more efficiently via FcγR engagement.^62^ In case of DENV infections, ADE is one of the several hypotheses proposed to explain the increase in disease severity associated with repeat heterotypic infections.^63, 64^

ADE of DENV infection has been demonstrated by multiple groups using sera and mAbs from primary and secondary dengue patients. Both neutralizing and non-neutralizing mAbs have been shown to greatly enhance DENV infection *in vitro.*^54, 56, 58, 60^ More recently, a few studies have also demonstrated that the ADE capacity of dengue-induced antibodies can also extend to ZIKV.^32, 48^ These studies are important from an epidemiological perspective, as the vast majority of regions that have reported ZIKV cases also experience DENV outbreaks. While timely, the findings of these studies are not entirely surprising, given the significant biological similarities and abundant epitopes shared between the two viruses. In one of these studies, EDE mAbs, shown to potently neutralize all four DENV serotypes^40^ as well as ZIKV,^48, 61^ also enhanced ZIKV infection *in vitro* by ADE. However, incubating neutralizing concentrations of specific EDE mAbs with enhancing concentrations of polyclonal dengue sera reduced infection of FcγR-bearing cells. In contrast, the presence of poorly neutralizing FLE mAbs did not abrogate the enhancement of infection by serum antibodies.^48^ These data suggest that the neutralization potential of antibodies targeting certain epitopes, such as EDE, may impede the ADE effect of enhancing antibodies, emphasizing the possible advantages of epitope-based vaccine design.

### Protective potential of cross-reactive antibodies

**Figure 3:**
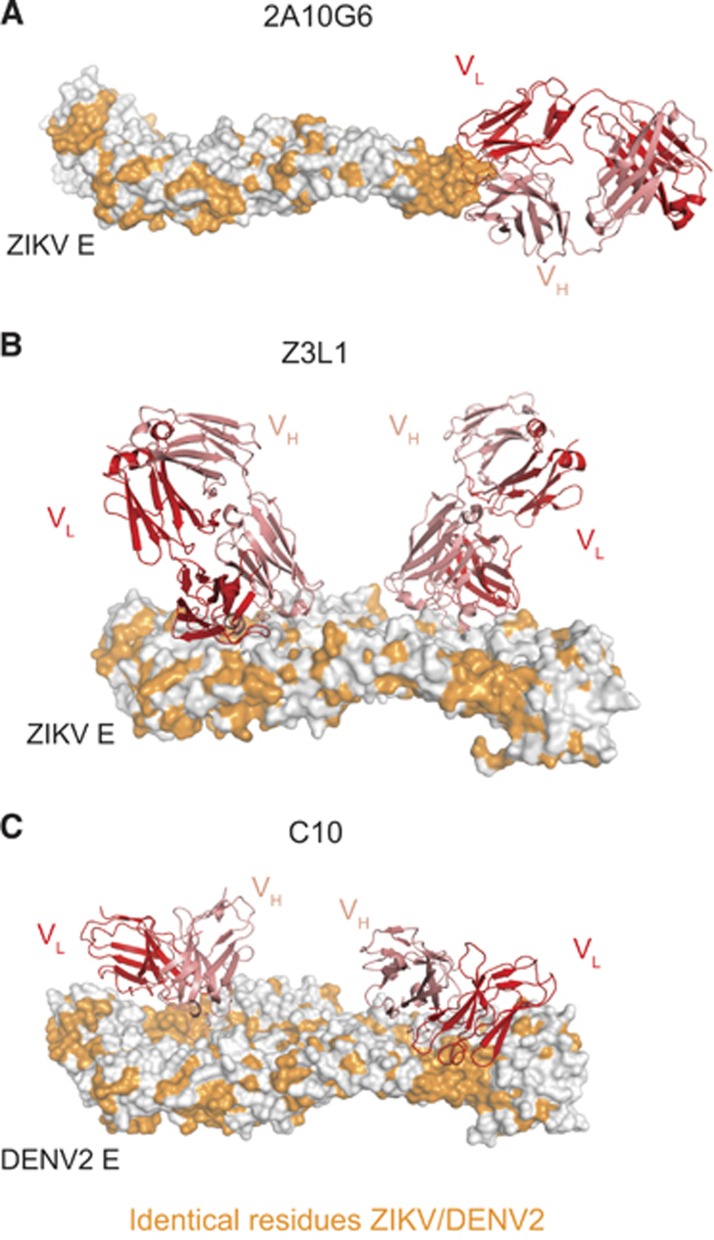
Cross-reactive and ZIKV-specific mAbs binding their E protein epitopes. (**A**) Murine mAb 2A10G6 binding to the conserved fusion loop on a ZIKV E monomer.^35^ (**B**) Human mAb Z3L1 binding to a ZIKV-specific EDI epitope.^45^ (**C**) Human mAb C10 (ribbon structure) binding to a dimer-dependent epitope on E protein dimer.^71^ For (**A–C**), heavy and light chains of mAbs are colored red and pink, respectively. All residues conserved between DENV2 and ZIKV are colored orange. All figures were created using PyMol (Schrödinger LLC).

In addition to the *in vitro* analyses described above, the protective capacity of several murine and human ZIKV-reactive mAbs has also been assessed *in vivo*. The fusion loop-specific murine mAb 2A10G6 (Figure 3A) was found to confer *in vivo* protection from ZIKV infection, albeit at a suboptimal dose of 500 μg.^35^ In another study, the EDIII lateral ridge-specific murine mAbs ZV-54 and ZV-67 protected mice from lethal challenge.^65^ Unlike 2A10G6, the EDIII mAbs were ZIKV-specific and did not bind to DENV *in vitro*. Such ZIKV-specific mAbs may possess a selective advantage over broadly reactive mAb in their inability to induce ADE of DENV infection.

The human MBC-derived mAb ZIKV-117 was evaluated for its prophylactic and therapeutic efficacy in a pregnant and non-pregnant mouse model. In addition to reducing mortality in wild-type adult mice, in the fetal transmission model, administering ZIKV-117 decreased placental injury, reduced ZIKV infection of placenta and fetal tissue and improved fetal outcome overall. Epitope mapping of ZIKV-117 suggested that the mAb binds a quaternary epitope on the E protein dimer–dimer interface.^46^ Another group generated a panel of mAbs from a convalescent ZIKV patient and of the mAbs isolated, Z23 and Z3L1 demonstrated potent ZIKV-specific *in vitro* neutralization and protected mice from weight loss and mortality after ZIKV infection. Although Z23 was mapped by cryo-EM to bind to EDIII, Z3L1 appeared to make contact primarily with EDI residues^45^ (Figure 3B). Unlike the above mAbs, EDE-specific mAb C10 (Figure 3C) was isolated from a DENV-experienced but presumably ZIKV-naive donor, and was also shown to protect mice from lethal challenge.^47^ Although these results are immensely promising, additional studies are required to dissect the mechanism of neutralization of these various mAbs. Moreover, several of these aforementioned studies were performed in immunocompromised mice, and therefore a more physiologically relevant characterization of their prophylactic and/or therapeutic potential merits further investigation in macaque models.

Sero-epidemiological data from historical ZIKV outbreaks as well as the findings of recent human serum-based studies may also provide added insight to the protective potential of immunological cross-reactivity against ZIKV infection. As demonstrated by *in vitro* studies, secondary dengue patient sera can strongly neutralize ZIKV while primary DENV sera exhibit limited cross-neutralization activity.^32, 47^ This could be explained by the differences in longevity of type-specific versus cross-reactive antibodies after DENV infection. Although type-specific responses after primary infection are believed to be long-lived, cross-protective immunity can wane months after infection. Secondary heterotypic DENV exposures, on the other hand, may boost cross-reactive antibody production by reactivating cross-reactive MBCs, potentially resulting in broader neutralization capacity. Additionally, while acute or early convalescent (≤100 days after symptom onset) secondary sera were shown to potently neutralize ZIKV, other studies showed that late convalescent sera exhibited poor-to-moderate ZIKV neutralization.^32, 47, 48^ These data suggest that while recently DENV-exposed individuals may maintain protective antibody titers against ZIKV, DENV-seropositive individuals exposed to ZIKV years after their dengue exposure may not benefit from effective cross-protection. These findings are consistent with the health outcomes of past and current ZIKV outbreaks. French Polynesia and Brazil, both countries with high DENV seroprevalence, experienced significant ZIKV epidemics with adverse clinical presentations. Cases of birth abnormalities and severe disease have also been reported in numerous DENV-endemic areas^28, 29, 66^ suggesting that prior DENV exposure may not protect against future ZIKV infections. Future studies comparing naive versus recall human responses may help clarify the role of pre-existing cross-reactive antibodies during ZIKV disease, shedding more light on the protective/pathological potential of DENV immunity in the context of ZIKV infection.
